# A Machine Learning Approach for Identifying Amino Acid Signatures in the HIV *Env* Gene Predictive of Dementia

**DOI:** 10.1371/journal.pone.0049538

**Published:** 2012-11-14

**Authors:** Alexander G. Holman, Dana Gabuzda

**Affiliations:** 1 Department of Cancer Immunology and AIDS, Dana-Farber Cancer Institute, Boston, Massachusetts, United States of America; 2 Department of Neurology (Microbiology, and Immunobiology), Harvard Medical School, Boston, Massachusetts, United States of America; University of Pittsburgh Center for Vaccine Research, United States of America

## Abstract

The identification of nucleotide sequence variations in viral pathogens linked to disease and clinical outcomes is important for developing vaccines and therapies. However, identifying these genetic variations in rapidly evolving pathogens adapting to selection pressures unique to each host presents several challenges. Machine learning tools provide new opportunities to address these challenges. In HIV infection, virus replicating within the brain causes HIV-associated dementia (HAD) and milder forms of neurocognitive impairment in 20–30% of patients with unsuppressed viremia. HIV neurotropism is primarily determined by the viral envelope (*env*) gene. To identify amino acid signatures in the HIV *env* gene predictive of HAD, we developed a machine learning pipeline using the PART rule-learning algorithm and C4.5 decision tree inducer to train a classifier on a meta-dataset (n = 860 *env* sequences from 78 patients: 40 HAD, 38 non-HAD). To increase the flexibility and biological relevance of our analysis, we included 4 numeric factors describing amino acid hydrophobicity, polarity, bulkiness, and charge, in addition to amino acid identities. The classifier had 75% predictive accuracy in leave-one-out cross-validation, and identified 5 signatures associated with HAD diagnosis (p<0.05, Fisher’s exact test). These HAD signatures were found in the majority of brain sequences from 8 of 10 HAD patients from an independent cohort. Additionally, 2 HAD signatures were validated against *env* sequences from CSF of a second independent cohort. This analysis provides insight into viral genetic determinants associated with HAD, and develops novel methods for applying machine learning tools to analyze the genetics of rapidly evolving pathogens.

## Introduction

The identification of nucleotide sequence variations in viral pathogens linked to disease and clinical outcomes is important for developing treatments and vaccines, and furthering our understanding of host-pathogen interactions. However, identifying viral mutations correlated to disease phenotype requires addressing a number of challenges, including high viral mutation rates and rapid evolution of viral pathogens in response to host selection pressures. Rapidly evolving viral pathogens, such as HIV, hepatitis C, and influenza, adapt to immune and drug selection pressures unique to each host as well as unique microenvironments within individual tissue sites [1]–[6]. Additionally, viral populations within a host often share phylogenetic lineages due to founder effects and genetic bottlenecks arising from primary infection by a small viral population [1], [7], [8]. Amino acid sequences exist within the three-dimensional structure of a folded protein, bringing distant regions in close proximity and increasing the likelihood of compensatory mutations and genetic covariation between non-contiguous amino acid positions [9]. Moreover, in some instances similar amino acids can fulfill similar biochemical roles within a protein, making them functionally interchangeable [10], [11]. Because of these properties, biologically relevant signatures have the potential to include sets of amino acids with similar biochemical properties at positions distant in the linear sequence. Addressing these challenges requires statistical methods able to mine complicated datasets and discriminate between relevant genetic signatures and patient-specific adaptations.

Recent works have applied machine learning tools to discover patterns in noisy biological datasets [12]–[14]. For example, classifier-based machine learning methods trained on HIV sequences can accurately predict biologically relevant outcomes such as coreceptor usage, immune epitopes, and drug resistance mutations, and identify functional groupings of amino acid positions within protein classes [11], [15], [16]. However, many of these works focus on development of a tool for classification of novel sequences, and thus utilize machine-learning algorithms, such as SVM, whose resulting classifiers are not easily interpretable [17]. Pillai et al. applied the more interpretable C4.5 and PART algorithms to investigate amino acid positions discriminating HIV coreceptor usage or tissue compartment of origin [4], [16], [18], though the positions identified were not used to generate sets of signatures correlated to a particular class or outcome. Further studies have identified genetically linked amino acid positions in the HIV *env* by utilizing mutual information analysis and evolutionary-network modeling [19]–[21]; however, correlation to clinical outcome was not explored. Recent work identified HIV *env* signatures found in early infection, but this analysis assessed participation in *a priori* defined structural and functional groups [22]. Current machine learning algorithms can train a naïve classifier to identify genetic signatures correlated with clinical outcome with no requirement for initial structural or functional information. However, careful algorithm selection and dataset assembly is required to allow interpretation of the resulting classifier.

The genetic diversity and high mutation and replication rate of HIV create significant opportunities and challenges for sequence analysis [23]. As well as being the causative agent in AIDS, HIV replicating in the brain is linked to development of HIV-associated neurological disorders (HAND) of which the most serious, HIV-associated dementia (HAD), occurs in 20–30% of untreated patients [24], [25]. Highly active antiretroviral therapy (HAART) has reduced the incidence of HAD, but the prevalence of less severe neurocognitive disorders has increased significantly [26]–[34]. Furthermore, in settings where access to antiretroviral treatment is limited, HAD remains a significant cause of mortality and morbidity [24].

The mechanisms leading to the development of HAD are not well understood (reviewed in [24], [32], [34], [35]). HIV enters the brain early in acute infection, likely via trafficking of infected lymphocytes and monocytes [36]–[39]. HIV replicates in CD4+ T-cells and macrophages in non-brain tissues and predominantly in macrophages and microglia within the brain [24], [40]–[42]. Neuronal injury may begin during the burst of viral replication occurring in the acute phase soon after infection, and may continue during chronic replication of virus in the brain throughout infection [24], [30]. However, the presence of virus replicating in the brain alone is not sufficient to induce neuronal damage; only a subset of patients develop neurocognitive impairment and there is disagreement over whether high levels of viral replication in the blood, CSF, or brain are predictive for development of HAD (reviewed in [24], [32]). Nadir CD4 count and baseline plasma or CSF viral load are associated with increased risk of neurocognitive impairment in treatment-naïve patients; however, these relationships are confounded by HAART treatment and tissue site variations in viral load [27], [43]–[50]. A better understanding of mechanisms underlying development of HAD is required for improved diagnosis, treatment, and prevention.

The HIV *env* gene is the main viral determinant of macrophage tropism and viral replication in the brain, and has also been implicated in viral neurotoxicity [24], [38], [51]–[57]. Potential causes of neurotoxicity include direct effects, including *env* binding and activation of chemokine receptors, or bystander effects, such as immune activation and inflammation [24], [35], [58]–[61]. Viral entry into the brain is thought to be ubiquitous across patients; however, levels of viral entry and replication in the brain vary from undetectable to high, as do degrees of neurocognitive impairment, though not necessarily in tandem [34], [45], [46], [49], [62]–[64]. Previous work demonstrated a close relationship between macrophage tropism and brain compartmentalization [65]–[69], and identified amino acid positions in *env* associated with replication in brain or development of HAD [18], [56], [70]–[75]; however, these findings are not sufficient to explain the observed clinical variability, nor do they address combined effects of multiple amino acid positions. To identify genetic signatures in the HIV *env* gene associated with HAD, we developed a machine-learning pipeline capable of mining genetic sequences to identify sets of amino acids correlated to clinical outcome. We then applied this pipeline to the analysis of a meta-dataset of HIV *env* sequence sampled from the brain of 78 patients clinically assessed for the development of HAD.

## Methods

### Ethics Statement

This study was conducted according to the principles expressed in the Declaration of Helsinki. The IRB at Dana-Farber Cancer Institute approved the research as exempt because all data and samples were obtained anonymously without any donor identities.

### Assembly of Training and Validation Meta-datasets

We utilized the HIV Brain Sequence Database [76], [77] to assemble a training meta-dataset of 860 clade-B HIV *env* sequences cloned directly from the brain of 78 patients without prior coculture or in vitro passage (Table 1). Clinical diagnoses of HIV-associated neurological disorders were obtained for all patients, either from the database or their original publications. In most cases diagnoses correspond to guidelines established by the Working Group of the American Academy of Neurology AIDS Task Force [78] and updated by the National Institute of Mental Health and the National Institute of Neurological Diseases and Stroke Working Group [25]. For each patient, genetic compartmentalization was assessed using the Slatkin-Maddison test [79], implemented in Hyphy [80], to make pairwise comparisons between tissue compartments with greater than 20 sequences in at least one compartment and greater than 4 sequences in both, as indicated by previous work benchmarking compartmentalization analysis [81].

**Table 1:** Summary of patients included in the brain training, brain validation, and CSF validation HIV env sequence datasets.

	HAD	non-HAD	All
**A** Brain training set			
Publications			18
Patients	40	38	78
Sequences	604	256	860
Median CD4^a^: count (range)	40 (2–400)	246 (0–824)	87 (0–824)
ART Treatment^b^	ART: 18, none: 2, unknown: 13	ART: 8, none: 16, unknown: 14	ART: 26, none: 2, unknown: 27
**B** Brain test set			
Publications			3
Patients	10	0	10
Sequences	75	0	75
Median CD4^a^: count (range)	60 (77–120)		60 (77–120)
ART Treatment^b^	ART: 3, none: 1, unknown: 6		ART: 3, none: 1, unknown: 6
**C** CSF			
Publications			7
Patients	27	14	41
Sequences	277	116	393
Median CD4^a^: count (range)	137 (16–592)	200 (13–512)	146.5 (13–592)
ART Treatment^b^	ART: 20, none: 3, unknown: 4	ART: 5, none: 2, unknown: 7	ART: 25, none: 5, unknown: 11

Patient annotations and publication references available in Table S1.

a Cells per microliter.

b ART, antiretroviral therapy.

### Generation of Phylogenetic Tree

An amino acid consensus sequence was generated for each patient using the consensus maker tool at the LANL HIV Sequence Databases [82]. Consensus sequences were used to generate a phylogenetic tree by maximum likelihood in the Treefinder program [83] using the JTT substitution matrix, and an optimized discrete Gamma heterogeneity model with 4 rate classes.

### Alignment, Weighting, and Translation of Sequences to Amino Acid Properties

Sequences were aligned and translated to amino acids using the HIVAlign tool from the LANL HIV Databases [82] implementing HMM-align [84], and the resulting alignments manually adjusted. Shannon entropy of the amino acid alignment was calculated using the Entropy-One tool hosted at the LANL HIV Sequence Databases [82]. To ensure that patients with different sequencing depth were weighted equally, individual sequences were weighted by: (total number of sequences in the dataset)/(total number of patients * total sequences in that patient) as described previously [85].

Four numeric factors describing amino acid biochemical properties were added to the alignment, in addition to amino acid identities, representing each amino acid position in each sequence as a categorical identity plus a vector of 4 numeric factors. These factors, representing amino acid polarity, secondary structure, molecular size or volume, and electrostatic charge, were derived in work by Atchley et. al. [10] by applying factor analysis to the 494 attributes in the AAIndex [86]. The resulting alignments of amino acid identities and factors were converted to ARFF format using a custom perl script.

### Generation of Signatures Sets Using the PART Algorithm in Weka

Weka version 3.7.3 was used as a data mining platform and included the J48 implementation of the C4.5 decision tree inducer [87]. Feature selection was performed using the WrapperSubsetEval method and J48 decision tree inducer both with default parameters for feature evaluation, and the BestFirst greedy hill-climbing algorithm for optimal feature search. For the selected features, the PART rule-learning algorithm [88] utilizing the J48 decision tree inducer was applied with default parameters to classify sequences by HAD diagnosis. Individual rules within the rule-set were interpreted as amino acid signatures. Rules based on numeric ranges of amino acid factors were converted to lists of matching amino acids. Accordingly, ranges of biochemical features derived by machine learning may include amino acid identities not actually observed at that position in the training dataset. Therefore, only those amino acids actually observed at that position within the training dataset are included in the signature. After signature generation, all amino acid positions included in signatures were removed from the original dataset and the feature selection and PART steps were iterated. Iterative signature generation continued until signatures gave no improvement over random class assignment as assessed by the kappa statistic < = 0, calculated using a multi-instance learning wrapper to account for grouping of sequences by patient.

### Validation

Leave-one-out cross validation was performed using a custom perl script, sequentially holding out one patient from the training set, retraining the classifier, and evaluating its ability to predict the class of the held out patient. Optimal patient classification was achieved using HAD signatures only. Patients were classified as HAD when 95% of their constituent sequences matched a HAD signature.

Fisher’s exact test was used to assess the distribution of HAD and non-HAD patients with sequences matching each signature. Q-values were calculated from p-values using fdrtool {Untitled:tn} in R.

## Results

### A Machine Learning Pipeline for Genetic Analysis

To develop an exploratory tool that would allow identification of HIV genetic signatures correlated to HAD, we developed a machine-learning pipeline utilizing the C4.5 decision tree inducer incorporated in the PART algorithm to analyze a meta-dataset of *env* sequences. A similar method utilizing the PART algorithm with cross-validation was previously used to predict coreceptor usage of HIV *env* sequences, resulting in greater predictive accuracy than the “charge rule” [16].

**Figure 1:**
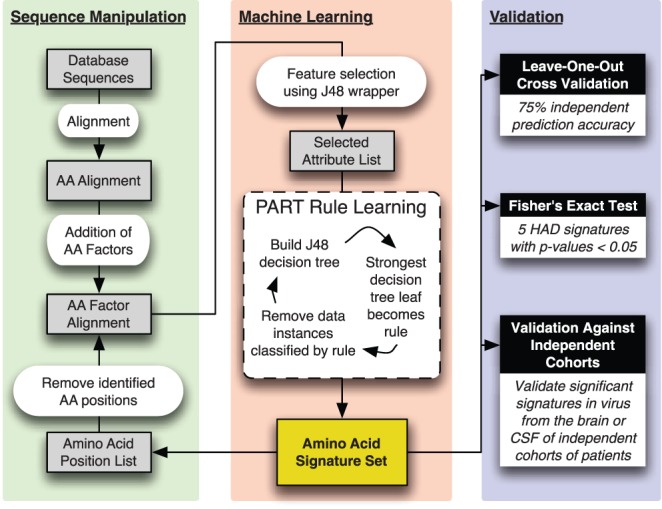
Analysis pipeline for identification and validation of genetic signatures associated with HAD. After initial assembly, alignment, and weighting of the sequence dataset, for each amino acid position in each sequence, four numeric factors describing the biochemical properties of the amino acid at that position are added to the alignment. This factor alignment enters the machine-learning phase where preliminary feature selection is used to select the attributes (amino acid identities or biochemical factors) that best differentiate between classes. Using the PART algorithm, this reduced set of attributes is used to train decision rules describing amino acid signatures correlated to disease outcome. Amino acid positions included in these signatures are removed from the main factor alignment and the process is iterated until no additional discriminatory signatures can be generated. Signatures are then validated by leave-one-out cross-validation, Fisher’s exact test, and assessment in brain and CSF-derived virus from independent cohorts.

Decision tree induction and specifically the C4.5 algorithm is a powerful method of training an interpretable classifier that identifies sets of attributes able to differentiate between classes of observations. The algorithm has the advantage of functioning well within a noisy dataset and incorporates methods of accounting for missing data, an important factor as differing sequencing coverage leads to incomplete data at borders of the region analyzed. The C4.5 algorithm trains a decision tree by sequentially adding attributes that best differentiate between class, then pruning the resulting tree to control for overfitting. The resulting decision tree is readily interpretable, allowing identification of sets of attributes most discriminatory for class. To translate decision trees into independent sets of genetic signatures, we adopted the PART algorithm, based on C4.5, to generate decision rule sets. Briefly, the PART algorithm uses C4.5 to generate a decision tree, and then interprets the path from the root of the tree to the strongest leaf as a rule set predictive of the class of that leaf. All sequences matching that rule are removed from the analysis, and the process is iterated to generate a new rule until all sequences can be classified. The PART algorithm has the advantage of generating sets of independent rules linked to class, each of which can be interpreted as an amino acid signature correlated to patient diagnosis. Using feature selection to reduce the number of attributes supplied to a machine-learning algorithm has been shown to improve performance [89]. Therefore, prior to rule generation using the PART algorithm, we used a wrapper method with the C4.5 decision tree inducer evaluated by a greedy hill-climbing algorithm to select the optimal set of attributes for machine learning. Because the PART algorithm generates an optimal classifier, not necessarily a classifier that captures the entirety of the structure within the data, we recursively applied PART, removing amino acid positions incorporated into signatures after each iteration, until the resulting classifier showed no improvement over random assortment by kappa statistic. The kappa statistic measures the chance-corrected agreement between the classifier and true classes; a kappa statistic greater than zero indicates better than random assortment and a kappa of one indicates perfect agreement [90]. The complete analysis pipeline is illustrated in Figure 1.

### Meta-dataset Assembly

One challenge to examining the viral genetics associated with development of HAD is assembling a dataset of brain-derived viral sequences containing a sufficient sample size of patients and sequences to provide statistical power for data analysis. The majority of brain tissue samples are obtained at autopsy, and few studies have assembled a large cohort of HIV patients and samples. To address this, we used the HIV Brain Sequence Database (HBSD) to assemble a meta-dataset containing published clade B HIV *env* sequences cloned from brain tissue [76], [77]. The HBSD is a curated database of HIV *env* sequences cloned directly from tissues, using methods that minimize the chance of PCR resampling. Previous work sequencing brain-derived *env* sequences has focused mainly on the V3 region, which contains important determinants of viral coreceptor usage, macrophage and brain tropism, and influences interactions with chemokine receptors, which in turn may influence neuroinflammation and neurotoxicity [24], [35], [52], [61], [91]–[93]. We focused on the V3 loop and surrounding C2 and C3 regions, amino acid positions 265–369 (numbered according to the reference strain HXB2, Genbank accession number K03455), both because of its biological importance and because this region provided the greatest number of patients and sequence depth. The meta-dataset contains 860 sequences from 78 patients (40 HAD and 38 non-HAD) (Table 1 and Table S1A). The majority of patients (n = 63) were sampled at autopsy with late-stage AIDS and low CD4 counts (median CD4 T cell count was 87 cells/µL); however, this dataset also included 15 patients with pre-symptomatic HIV infection who died of non-AIDS related causes. The majority were sampled between 1991 and 2000, and were drug naive or on pre-HAART regimens. For all patients with sufficient sequences from brain and non-brain tissue sites for phylogenetic testing of compartmentalization (38 of 78 patients), brain-derived sequences were genetically compartmentalized from non-brain sequences (p<0.05 by Slatkin-Maddison test for compartmentalization). Patients were clinically assessed for dementia status and grouped either as HAD, which included diagnoses of mild, moderate, or severe HAD and severity not specified, or non-HAD, which included patients that were clinically assessed and determined to be non-demented. Removing mild-HAD patients from the analysis, including only severe-HAD patients, or removing presymptomatic patients, produced similar results to analysis of the full dataset, albeit at lower statistical power due to reduced patient numbers (data not shown).

Within the meta-dataset, patient depth of sequencing was variable, ranging from patients with a single sequence to patients with 116 sequences. Median sequencing depth was 5, and was similar between HAD and non-HAD (5.5 and 5, respectively). Virus within each patient is genetically related due to founder effects caused by infection by a small initial population. Unaddressed, this has the potential to bias analysis for motifs found in highly sequenced patients. To account for this effect, we weighted sequences by the inverse of patient sequencing depth, such that all patients had equal weight during data mining, as described previously [85].

**Figure 2:**
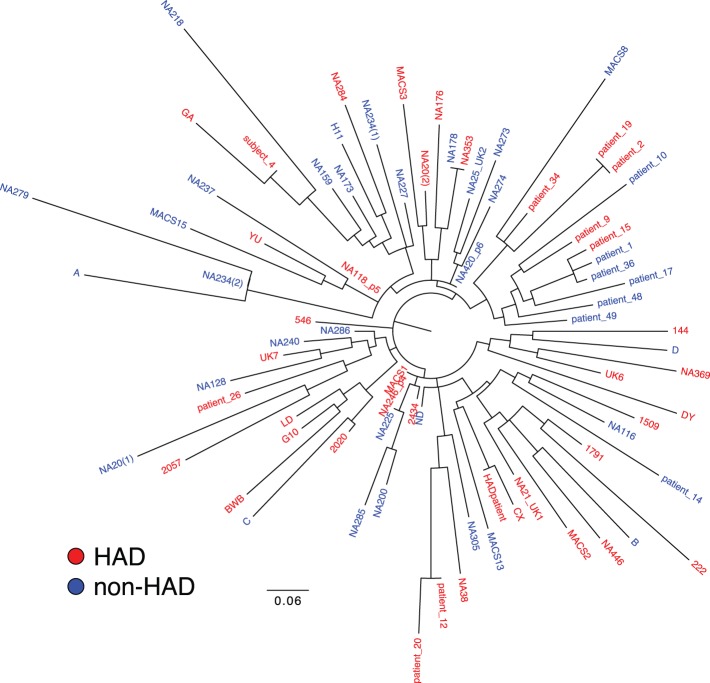
Unrooted phylogenetic tree of patient consensus sequences for the C2-V3-C3 region of HIV *env.* A consensus sequence for the C2-V3-C3 region was generated for each patient in the brain dataset (n = 78). These consensus sequences were used to generate an unrooted maximum likelihood tree. Patients are colored by HAD diagnosis and identified by patient codes taken from their original publication.

To rule out patient clustering by study or tissue bank, or transmission chains, we constructed a phylogenetic tree of the amino acid consensus sequences for the C2-V3-C3 region of each patient (Figure 2). We observed no patient clustering by study or tissue bank. Additionally, we observed no clustering by dementia status; HAD (red) and non-HAD (blue) patients were interspersed throughout the branches of the tree.

### Addition of Biochemical Factors to the Amino Acid Alignment

A preliminary analysis of the amino acid alignment identified positions where multiple amino acid identities were correlated to one class of disease outcome. Sets of a

mino acids can have similar biochemical properties and may fulfill the same functional role in a protein. To increase the flexibility and power of the analysis, we incorporated numeric measures of amino acid biochemical properties into the analysis. We utilized the work of Atchley et al. [10], which applied factor analysis to summarize the contents of the Amino Acid Index, a comprehensive but highly redundant database of 494 numeric descriptors of amino acid biochemical properties, into 5 global factors: Factor 1: Polarity, Accessibility, Hydrophobicity; Factor 2: Propensity for Secondary Structure; Factor 3: Molecular Size; Factor 4: Codon Composition; Factor 5: Electrostatic Charge. These factors are linear and numeric, allowing for integration into the machine-learning pipeline. We chose to include 4 of the 5 factors describing basic amino acid biochemical properties, excluding codon composition, as we were most interested in mining functional roles of amino acids within *env*. Our final dataset consisted of an amino acid alignment for which each position in each sequence consisted of a categorical attribute for amino acid identity and 4 numeric attributes describing properties of that amino acid. Analysis of identities plus 4 amino acid factors showed improvement in the descriptive power of resulting signatures over analysis of identities alone. At positions where multiple amino acids were correlated with class, mining identities alone resulted in generation of redundant signatures differing only at one position (data not shown). In contrast, the addition of numeric factors describing biochemical properties allowed generation of single signatures that included a numeric range encompassing correlated amino acids.

### Generation and Validation of Amino Acid Signatures

Analysis of the training dataset of amino acid identities and 4 biochemical factors completed 2 iterations of the data-mining pipeline, generating kappa statistics of 0.332 and 0.28. The pipeline stopped after the second iteration, discarding the third set of signatures with a kappa statistic of -0.14, indicating no improvement over random assortment. These first 2 iterations produced sets of 8 and 10 signatures, respectively. A negative control set was generated by randomly permuting HAD diagnosis class labels across patients. The data mining pipeline identified no predictive signature sets from the negative control set and stopped in the first iteration with a kappa value of −0.041 (data not shown). We retained the rules generated from this negative control for use in further validations. Because our dataset was not of sufficient size to split into training and test sets, we used leave-one-out cross validation to determine predictive accuracy and test for overfitting. This method generates a series of independent training and test sets by sequentially removing one patient from the training set, retraining the classifier and testing the ability of the classifier to predict the HAD status of the held-out patient. We examined the distribution of HAD and non-HAD classified sequences within patients to select criteria for patient classification. For the majority of patients, the percentage of sequences matching a signature was close to a binary division; either no sequence matched or all or nearly all sequences matched. HAD signatures were a stronger predictor of patient class than non-HAD signatures, and the most accurate predictions were made based on HAD signatures alone. This led us to empirically set the threshold for classifying a patient as HAD at 95% of the patient’s sequences predicted as HAD, yielding a 75% predictive accuracy in leave-one-out cross validation.

To assess the associations of individual signatures with patient class, we used Fisher’s exact test to evaluate the distribution of matching sequences across patients. The PART algorithm utilizes a layered approach to mine sub-structures within the data, removing matching sequences before training the next signature. However, we wished to determine which signatures were independently significant, outside the background of preceding signatures. Thus, we evaluated the distribution of matching sequences across patients in the complete brain dataset. 5 of 8 signatures in the first iteration and 5 of 10 signatures in the second iteration had p-values <0.05 (Table 2). Of these 10 signatures, 5 were associated with HAD and 5 with non-HAD. False discovery rate-adjusted q-values were significant (q<0.05) for each of these 10 signatures (data not shown).

**Table 2:** Statistical validation against patients in the brain HIV envsequence dataset of all HAD and non-HAD signatures generated by the PART algorithm.

Signature	Diagnosis	Patient Count:	Matching Patients:		p-value
		Total (HAD/None)	HAD	non-HAD	
1_01 *	HAD	77 (39/38)	10	0	1.0E-03
1_02 *	non-HAD	77 (39/38)	1	9	6.8E-03
1_03 *	non-HAD	77 (39/38)	2	8	0.047
1_04 *	HAD	77 (39/38)	18	1	7.5E-06
1_05	non-HAD	77 (39/38)	7	12	0.19
1_06	non-HAD	51 (31/20)	11	5	0.54
1_07 *	non-HAD	77 (39/38)	9	23	1.2E-03
1_08	HAD	77 (39/38)	34	33	1
2_01 *	HAD	77 (39/38)	9	1	0.014
2_02 *	non-HAD	76 (38/38)	0	9	2.3E-03
2_03 *	HAD	77 (39/38)	14	2	1.4E-03
2_04 *	non-HAD	70 (33/37)	9	20	0.030
2_05 *	HAD	76 (38/38)	25	4	1.0E-06
2_06	non-HAD	49 (30/19)	13	8	1
2_07	non-HAD	77 (39/38)	4	8	0.22
2_08	non-HAD	77 (39/38)	18	16	0.82
2_09	HAD	77 (39/38)	12	5	0.098
2_10	non-HAD	77 (39/38)	23	25	0.64

The statistical significance of all HAD and non-HAD signatures was determined using Fisher’s exact test to evaluate the distribution of patients in the brain dataset with matching sequences. Diagnosis indicates whether the signature was predictive of HAD or non-HAD. Patient count reflects the total number of patients with sequence spanning the amino acid positions in the relevant signature (i.e. signature 1_01 was tested in 77 patients because 1 patient does not contain sequences spanning positions 304 through 343, which are included in signature 1_01). The number of HAD and non-HAD patients from the brain dataset, containing sequences matching each signature are given, followed by the p-value of that patient distribution, calculated by Fisher’s exact test.

* = p-value <0.05.

One caveat to the analysis by Fisher’s exact test is that the p-values generated resulted from testing the frequency of these signatures in the same dataset from which they were generated. To examine this bias, we applied Fisher’s exact test to the set of negative control signatures generated in the first iteration (kappa value −0.041) of the patient class-permuted negative control described above. This test identified no signatures with p-values <0.05 (data not shown), indicating that the significant p-values we observed are unlikely due to applying Fisher’s exact test against our training dataset.

**Figure 3:**
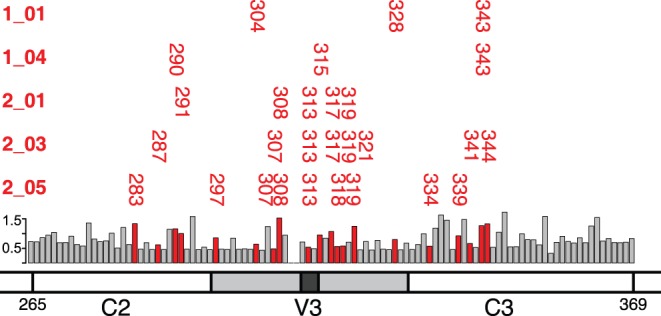
Amino acid positions identified in each HAD signature. Amino acid positions are plotted for each HAD signature against a schematic of the HIV C2-V3-C3 region examined. Shannon entropy values of all positions in the alignment are plotted as a bar graph, with colored bars marking positions included in HAD signatures.

**Figure 4:**
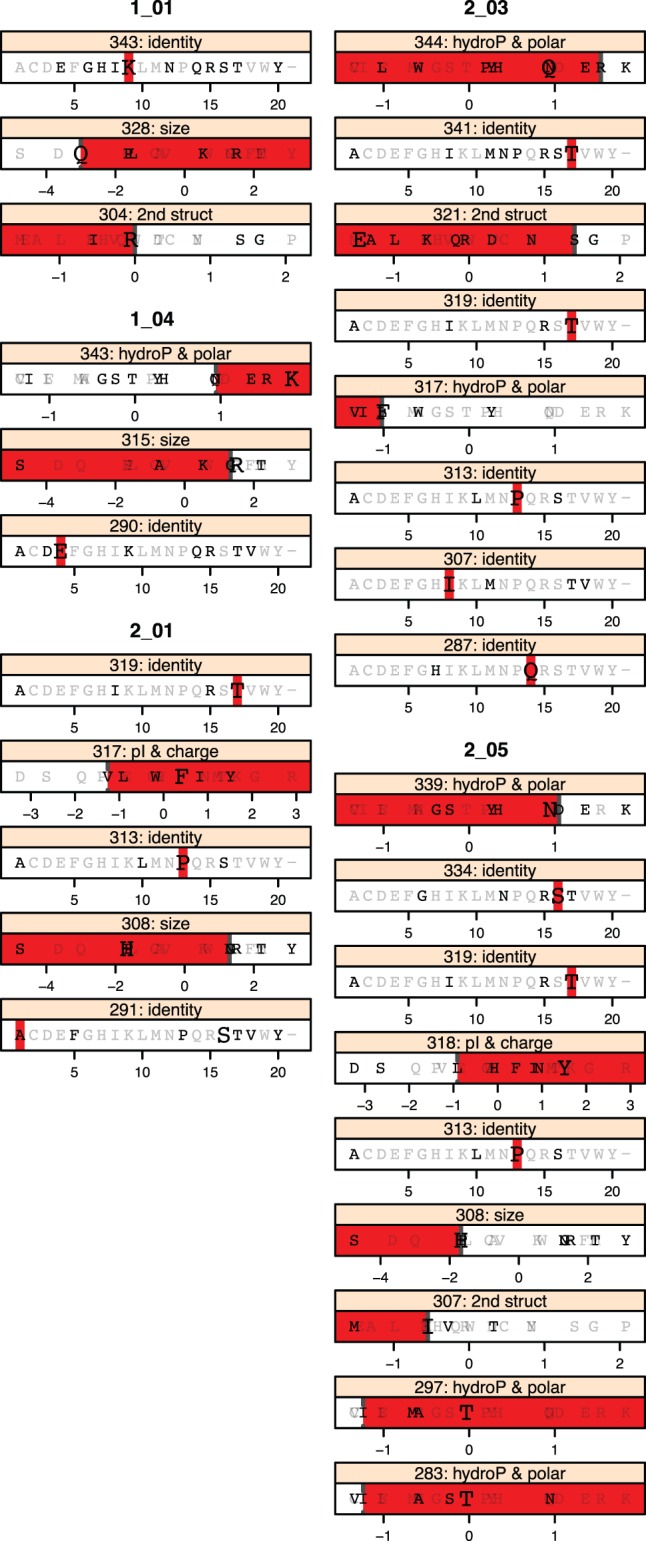
Amino acid identity and biochemical factor requirements for HAD signatures. Amino acid requirements at each position are plotted. For each “position: factor” pair, all amino acids are plotted at their value for that factor. Amino acids observed at that position within the brain-derived dataset are plotted in black, while those not observed are gray. The B-clade consensus amino acid is plotted in large font. The colored bar indicates the range of acceptable values in that signature. Lower range ends are open, indicated by a dotted line, (signature 1_01, position 328 excludes Q). Upper range ends are closed, indicated by a solid line (signature 2_03, position 321 includes S).

**Figure 5:**
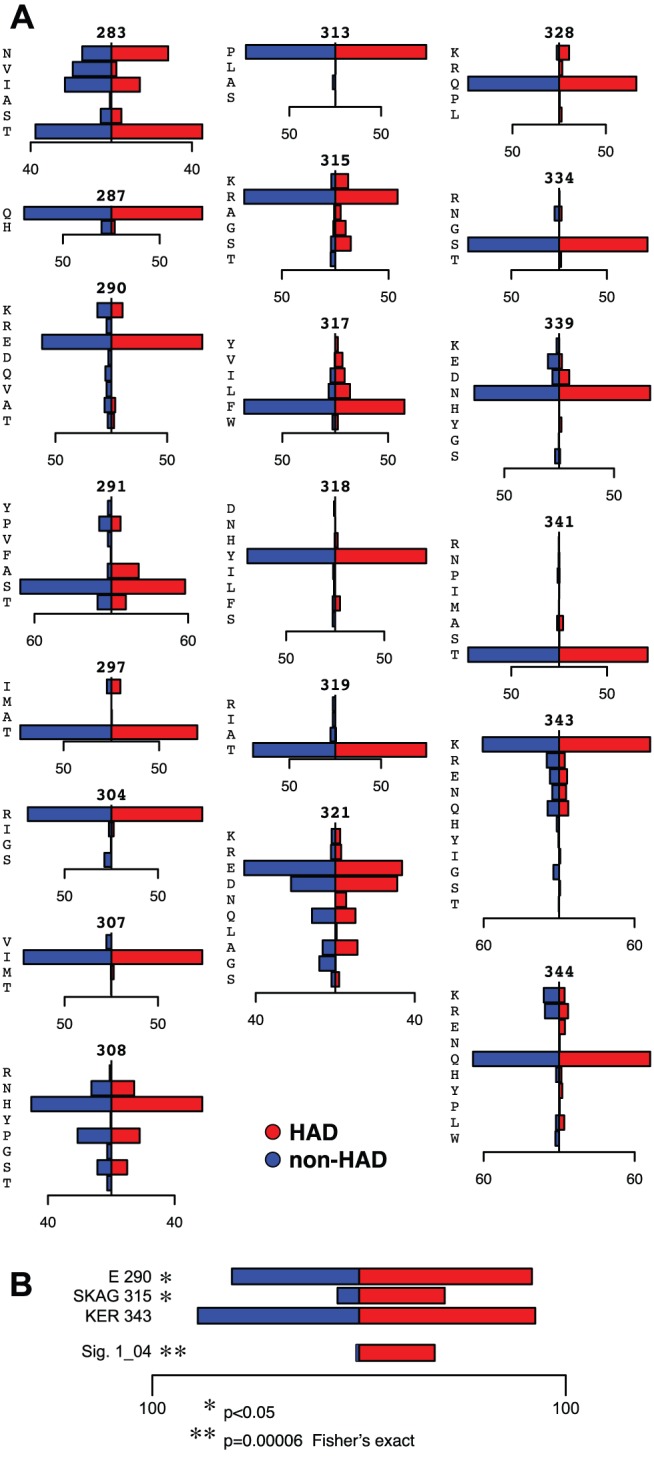
Amino acid distributions at individual positions are not correlated with HAD. A. Amino acid frequencies in the brain dataset plotted as distributions totaling 100% for each class (HAD, non-HAD). The weights of individual sequences are normalized by patient sequencing depth. B. Percentage of sequences of each class (HAD, non-HAD) matching the amino acid requirements of signature 1_04 at each position individually, and for the complete signature. Bars represent only matching sequences and thus do not sum to 100%.

Because initial analysis during cross-validation indicated that HAD signatures alone were the best predictor of patient class, we focused further analysis on the 5 HAD signatures that showed significant association with HAD diagnosis by Fisher’s exact test. Most of these signatures consisted of amino positions in the tip of the V3 loop and pairs of positions, approximately equidistant on either side of the tip of the V3 loop, spanning the C2-V3-C3 region (Figure 3). Shannon entropy was calculated for amino acid positions across the region analyzed. Signatures included both high and low entropy positions, demonstrating no clear bias by entropy. Examining the rules comprising each signature revealed that the amino acid requirements could consist of a combination of single amino acid identities, groups of amino acids, and larger amino acid sets (Figure 4 and Figure S1). Given the amino acids observed within the dataset, many of these larger amino acid sets effectively exclude a single amino acid. For example, within signature 1_01 position 328 includes a large range of amino acids based on size, which can be interpreted as a “not-Q” requirement. Unexpectedly, the amino acid distributions at individual positions did not demonstrate significant bias by HAD diagnosis (Figure 5). For example, the individual positions comprising signature 1_04 (290, 315, and 343) show only minor amino acid bias between HAD and non-HAD patients. Additionally, at each position the sets of amino acids from signature 1_04 show only a minor bias between HAD and non-HAD patients. KER at position 343 shows no significant bias and E at position 290 and SKAG at position 315 each have a p-value <0.05, but are not strongly associated with HAD. Instead, these positions only correlated to HAD diagnosis when combined into the overall signature.

**Figure 6:**
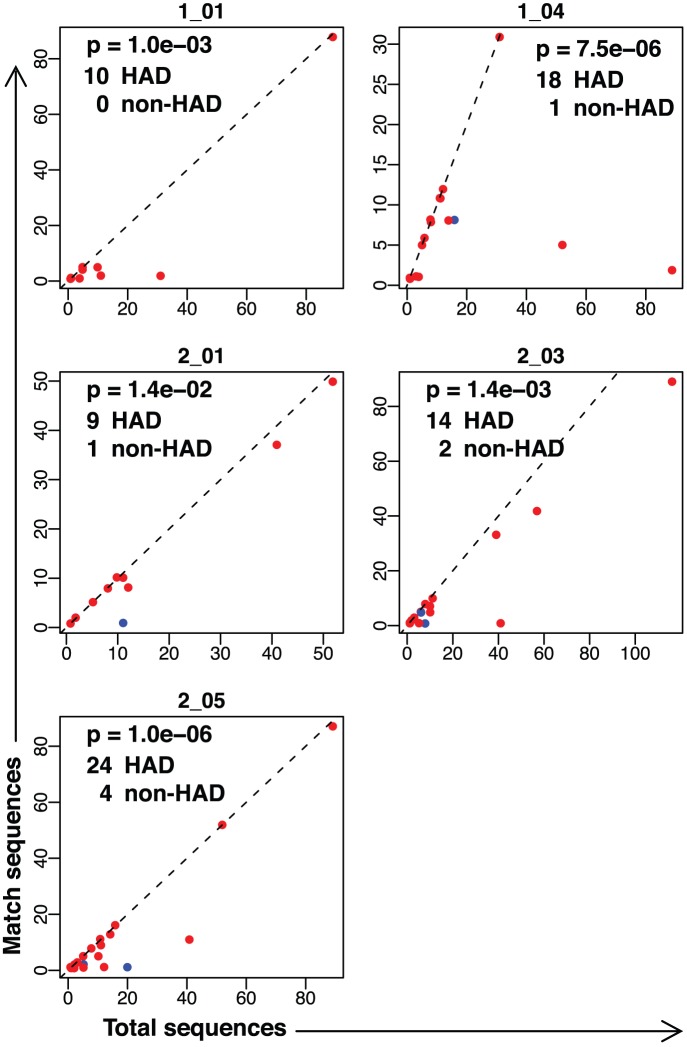
Proportion of sequences per patient from the brain dataset matching HAD signatures. For each HAD signature, HAD (red) and non-HAD (blue) patients are plotted according to their total number of sequences (x-axis) and the number of sequences matching the signature (y-axis). Patients with no matching sequences are omitted from the plot for clarity, but are included for statistical calculations. Dashed line indicates slope = 1 at which all sequences in a patient match signature. Jitter has been added to visualize overlapping points. Text indicates p-value by Fisher’s exact test and the number of patients from each class with matching sequences.

To better understand the distribution of each signature within patients, we visualized the proportion of sequences in each patient matching each of the significant HAD signatures (Figure 6 and Figure S2). Patients with no matches were omitted from the visualization for clarity, but were included in the statistical analysis with Fisher’s exact test. Signatures had a strong bias to uniquely match sequences derived from either HAD patients (Figure 6) or non-HAD patients (Figure S2). In addition to being highly discriminatory for patient class, matching sequences appear to have expanded nearly to fixation within patients. For most patients with sequences matching a signature, all or nearly all sequences were matching. Depth of patient sequencing seemed to have little effect on the likelihood of patient matches to a signature. In most cases, the proportion of matching sequences remained similar across patients with differing sequencing depth.

**Figure 7:**
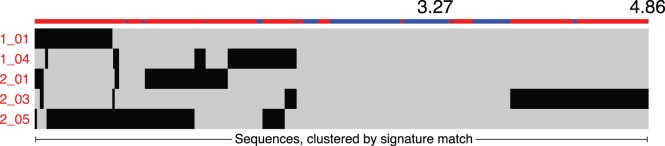
Distribution of matching sequences across HAD signatures. Visualization of sequences (x-axis) matching HAD signatures (y-axis). Colored bars on top of the x-axis indicate HAD (red) or non-HAD (blue) diagnosis of the patient from which the sequence was sampled. Sequences are clustered by their pattern of signature matches.

Sets of signatures can contain unique or overlapping amino acid positions and requirements, raising the possibility of individual sequences matching multiple signatures. To examine the propensity of sequences to match multiple signatures, we visualized individual sequences across all signatures (Figure 7). Several sequences matched 3 signatures; however, most sequences matched two or fewer signatures.

### Evaluation of Signatures in Two Independent Non-brain Datasets

**Figure 8:**
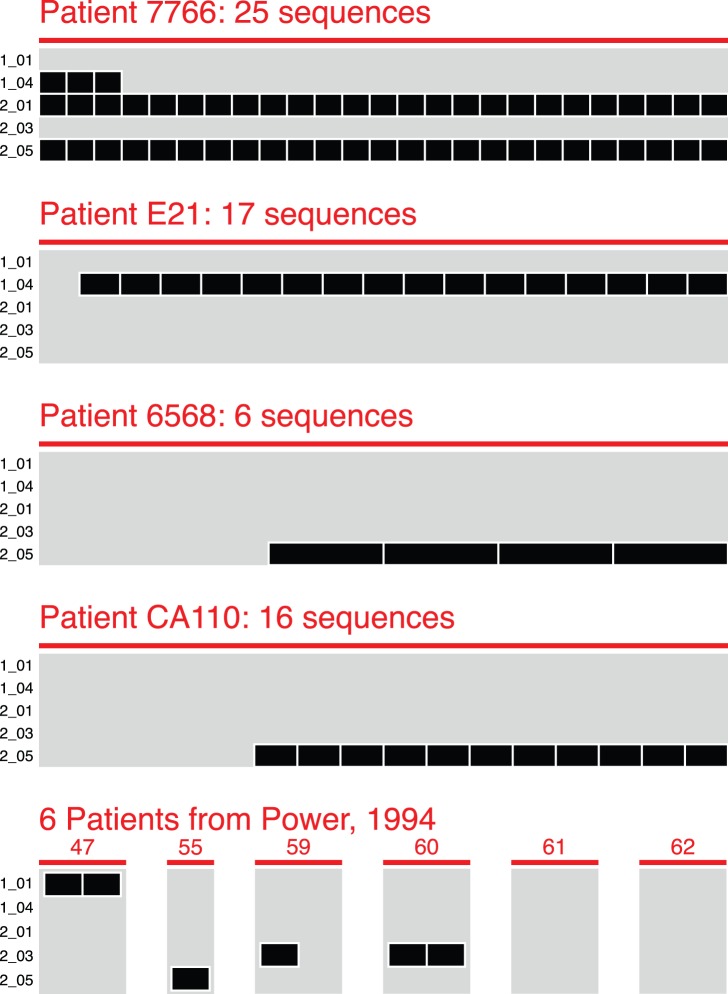
Validation of HAD signatures against brain-derived *env* sequences from an independent cohort. A total of 75 brain-derived sequences from 10 independent patients (x-axis) are visualized as matching or not matching each HAD signature (y-axis). All patients in the independent cohort were diagnosed with HAD. One sequence has been omitted from patient E21 because phylogenetic mapping from the original publication indicated it might be a blood-derived contaminant. A second sequence in E21, matching no HAD signatures, was of indeterminate compartment of origin, and was retained.

To assess these signatures in HIV *env* sequences from an independent cohort of patients, we assembled two validation datasets. The first consisted of virus sampled from the brain of 10 independent HAD patients (Table 1B and Table S1B). This dataset allowed us to empirically observe the occurrence of HAD signatures in sequences from the brain of an independent cohort, albeit one of insufficient sample size for statistical assessment, and containing only HAD patients (Figure 8). For most patients in this set, the majority or all sequences matched a HAD signature. For patient 7766, all 25 sequences matched signatures 2_01 and 2_05. All sequences from patients 47, 55 and 60 matched signatures 1_01, 2_05 and 2_03, respectively, though each of these patients is represented by only 1 or 2 sequences. Finally, the majority of sequences from patients E21, 6568 and CA110 matched signatures 1_04 and 2_05. Thus, 8 of 10 HAD patients from an independent cohort had 50% or greater sequences matching a HAD signature.

**Figure 9:**
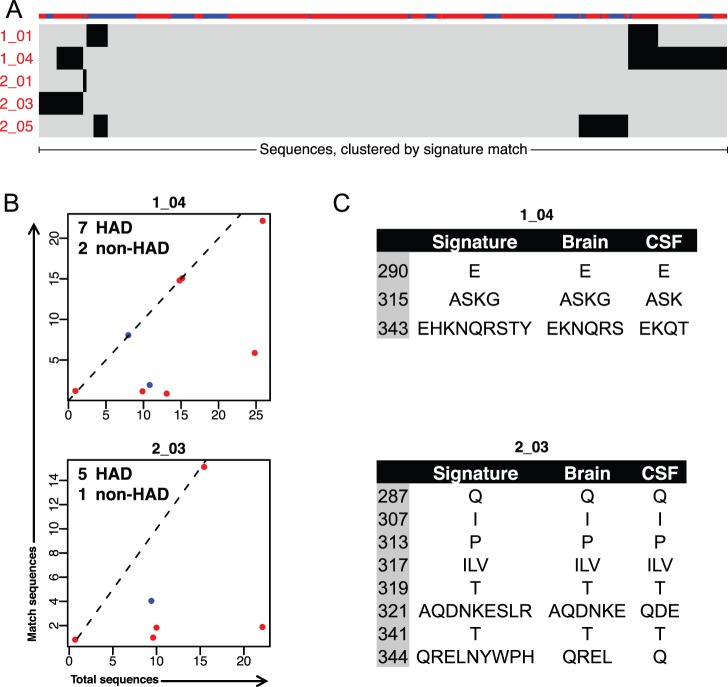
Validation of HAD signatures against CSF-derived sequences from an independent cohort. A. Visualization of 393 CSF-derived sequences from 41 independent patients matching HAD signatures. Conventions are the same as in Figure 7. B. Proportion of patients with CSF-derived sequence matching signature 1_04 and signature 2_03. Conventions are the same as in Figure 6. C. Amino acid diversity in sequences matching signature 1_04 and signature 2_03. Left column: amino acid requirements in the signature. Middle and right column: amino acids observed in matching sequences from the brain and from CSF.

The second validation set consisted of virus sampled from the CSF of patients clinically assessed for HAD diagnosis. In this case, we utilized CSF-derived virus as a surrogate for virus replicating in the brain, based on phylogenetic evidence that brain and CSF-derived virus are more closely related to each other than to non-CNS tissue sites [6]. The CSF-derived validation dataset consisted of 393 HIV *env* sequences cloned from the CSF of an independent cohort of 41 patients (Table 1 and Table S1C). 30 patients had reported CD4 T cell counts (median 147 cells/µL; range, 13–592), of which 23 had advanced disease (defined as current or nadir CD4 count <200). Patient CD4 counts, AIDS progression, and treatment histories were matched as closely as possible to patients in the brain-derived dataset and all virus was clade-B. HIV in the CSF can originate either from the brain or from blood and lymphoid tissues. Early in infection, virus in the CSF appears predominately blood and lymphoid-derived [94]; during late infection, phylogenetic analysis demonstrates that CSF virus is more closely related to virus replicating in the brain [69], [73], [95]. To increase the probability that the validation dataset was more likely brain-derived, we included only patients for which CSF-derived virus was genetically compartmentalized from virus sampled from non-CNS sites as determined by the Slatkin-Maddison test (p<0.05). Testing significant HAD signatures identified from the brain-derived dataset against sequences from the CSF demonstrated that HAD signatures 1_04 and 2_03 were predominantly matched by CSF virus from HAD patients (Figure 9A). Signature 1_04 matched 7 HAD and 2 non-HAD patients, and signature 2_03 matched 5 HAD and 1 non-HAD patient (Figure 8B), however, because of the small and unequally distributed number of patients in this dataset, these distributions did not reach statistical significance. Additionally, between brain and CSF derived datasets, sequences matching these signatures had similar diversities of amino acids (Figure 8C). Signature 1_04 requires ASK or G at position 315, matching sequence from the brain contains ASK and G, and matching sequence from the CSF contains AS and K.

## Discussion

Here we developed a method of applying machine learning tools to identify genetic signatures in viral pathogen genomes correlated to disease outcome, in this case the development of HIV-associated dementia. Our method expands the flexibility and biological relevance of the analysis by including numeric factors describing amino acid biochemical properties. We applied this method to the C2-V3-C3 region of the HIV *env* gene and identified 5 HAD signatures correlated to the presence of dementia. We evaluated these signatures in two independent datasets, and observed HAD signatures in the majority of brain-derived *env* sequences from 8 of 10 patients with dementia, and validated 2 signatures in HIV *env* sampled from the CSF. This work demonstrates that our machine-learning pipeline can identify biologically relevant genetic signatures in a noisy, real-world dataset of sequences from a rapidly evolving viral pathogen.

The identified amino acid signatures recapitulate and expand on previously published amino acid variants associated with HAD. Dunfee et al. 2006 reported that the N283 variant was present at high frequencies in virus from the brain of HAD patients, and increases gp120 affinity for CD4, enhancing replication in macrophages and microglia [71]. HAD signature 2_05 requires a polar, hydrophilic amino acid at position 283, which includes N, the most polar amino acid observed in that position in the brain dataset, as ranked by Atchley Factor I. Non-HAD signature 2_02 requires a V at position 283, the least polar most hydrophobic amino acid observed at that position in the brain dataset. Power et al. 1994 reported positions 305 and 329 correlated to HAD [56]; when converted to HXB2 numbering, these correspond to positions 308 and 333. At position 308, H was HAD associated, whereas P was non-HAD associated. Position 308 occurs in HAD signatures 2_01 and 2_05, in both cases requiring small amino acids. H is slightly smaller than P by Atchley Factor 3. Signature 2_05 includes H and excludes P, while signature 2_01 includes both, excluding larger amino acids. Position 333 was not identified in signatures; however, neighboring position 334 is found in signature 2_05. Pillai et al. 2006 identified positions 300, 304, 308, and 314 as associated with CSF versus blood, and S at position 300 in CSF virus associated with HAD [18]. Positions 304 and 308 were each included in several signatures we identified. Position 314 was not included, however, flanking positions 313 and 315 were found across 4 signatures. This also highlights one advantage to our approach; in addition to identifying positions with clear amino acid biases, we also identify linked sets of positions, only correlated when considered together.

Examination of the observed signatures within the structure of the HIV *env* protein suggests how these amino acids may interact within the three-dimensional structure of the active protein. The *env* V3 region consists of a stem-loop structure formed by amino acid positions 296 through 330, with positions 312 to 315 forming the tip of the loop. Most signatures incorporate a central position at the V3 loop tip flanked by pairs of equidistant positions on the stem, in agreement with previous work finding that covarying positions in V3 tend to bridge opposite strands of the V3 loop [19]–[21]. Signature 2_01 includes positions 308 and 317, previously described as linked [19], and signatures 2_03 and 2_05 contain other nearby positions (307 or 308, paired with 317 or 319). Other signatures follow a similar structural pattern, in some instances bridging greater distance between sites. Signature 1_01 includes positions 304 and 328, and signature 1_04 includes 290 and 343 in the C2 and C3 regions. Additionally, clusters of several positions appear to define important regions within the protein linked to HAD phenotype. Positions 343–344 occur in three HAD signatures, and positions 307–308 and 317–319 occur in three HAD signatures. Notably absent are positions in the conserved base of the V3 loop, (positions 298–303, and 322–327) [96]–[98], which is involved in interactions with the CCR5 coreceptor [99]. The amino acid properties required at each position may also begin to define functional requirements within the protein. Signatures 2_01 and 2_05 require low molecular size and low secondary structure at positions 307 and 308, while signature 2_03 requires isoleucine at position 307, which would also satisfy the size and secondary structure requirements of 2_01 and 2_05. These signatures also require hydrophobic, nonpolar residues with high pI and charge at positions 317–319.

Interpreting mechanistic implications of these signatures requires consideration of viral replication dynamics and how this could influence development of HAD. Low nadir CD4 counts and high baseline plasma viral load increase the risk of developing neurocognitive impairment in treatment-naïve patients [48] and suppressive HAART is protective, particularly against the more severe forms of HAD [27], [30], [34]. Nonetheless, brain atrophy can be detected by neuroimaging even in patients with well-controlled viral replication [26]. Though these results suggest an association with disease progression, HAD appears more closely linked to other mechanisms including chronic viral replication in the brain and activation of CNS macrophages and microglia. Previous reports have shown that HAD is associated with increased viral genetic compartmentalization in CSF compared to non-CNS tissues, suggesting that CSF viral sequences are derived from unique viral populations replicating within the brain [69], [73], [100]. The signatures we identified may be directly or indirectly related to these changes in CNS replication dynamics. In the first case, amino acid changes in the signatures may enhance HIV interactions with CD4 and/or CCR5, thereby increasing viral entry and replication in macrophages, viral replication in the brain, and/or neurotoxicity, possibly via increased immune activation or activation of chemokine receptors [58], [60], [61], [66], [69], [101]–[106]. Alternatively, signatures linked to HAD may reflect specific viral adaptations driven by host selection pressures, such as humoral or cellular immune responses targeting specific viral epitopes, that may differ between HAD and non-HAD patients. Further studies are needed to investigate these potential links between viral genetics and susceptibility to HAD.

Examination of the frequency of the identified signatures across patients showed that for most signatures, all or nearly all brain-derived viral sequences from a matching patient matched the signature. Most patients were sampled at autopsy with late-stage AIDS, allowing viral mutations conferring a selective advantage to expand to a majority variant. Further study incorporating sampling at earlier time points, for example longitudinal CSF samples with a brain sample obtained at autopsy, would better describe the dynamics of the emergence of viral genetic signatures and their role in development of HAD. Examining the pattern of matching sequences across signatures demonstrated that the dataset did not contain broadly matching viral sequences (Figure 7). Instead, we observed that sequences predominantly matched a small number of signatures, suggesting that the dataset consists of distinct subpopulations.

The method employed by the PART algorithm, iteratively generating a signature then removing sequences matching that signature, additionally has the potential to reveal interesting substructures within the dataset. In this study, we were interested in the distribution of signatures across all sequences in the dataset. However, we also performed a layered analysis by Fisher’s exact test, mirroring the PART algorithm by sequentially analyzing each signature and removing each sequence once it matches a signature (data not shown). By this approach we observed two additional HAD signatures, 1_08 and 2_09, with p-values <0.05 (Figure S1). These two signatures both demonstrated a dramatic shift in patient distribution between the independent and layered analysis. Evaluated independently, signature 1_08 was found in 34 HAD patients and 33 non-HAD patients (39 HAD and 38 non-HAD patients total), whereas by a layered approach, signature 1_08 is found in 16 HAD patients and 0 non-HAD patients (17 HAD and 5 non-HAD patients total). HAD signature 1_08 is relatively promiscuous alone, matching sequences from both HAD and non-HAD patients. However, the majority of non-HAD sequences matching 1_08 also match non-HAD signatures 1_02, 1_03, 1_05 and 1_07. When signatures are considered sequentially, removing sequences matching earlier signatures, the remaining 1_08 matching sequences are uniquely from HAD patients. This suggests that the amino acid changes in signatures 1_08 and 2_09 may represent a sub-pattern in the dataset, only linked to HAD in the absence of dominant changes from earlier signatures. Though further work is required to support these conclusions, this effect illustrates the power of the PART algorithm to uncover subsets of structure within the dataset.

We acknowledge some limitations of the study. As with any machine learning-based work, overfitting (training a classifier on random noise instead of true features correlated to outcome) is a concern we sought to address throughout study design. The C4.5 algorithm was selected in part because it incorporates a pruning step designed to remove overfit decision tree branches. The genetic relatedness of sequences within a patient was addressed by weighting individual sequences to normalize patients by sequencing depth. Finally, leave-one-out cross-validation generating independent training and testing sets and class-permuted negative controls were used to test for overfitting. Ideally, a machine learning analysis would initially divide the dataset into well balanced training and test sets. In this case, however, though 78 patients represents a dataset of unprecedented size in the field, it was not of sufficient size to split for data mining, requiring the use of cross-validation methodologies. We were, however, able to assemble an independent test set of 10 HAD patients, in which we validated the predictive power of these signatures.

Application of this pipeline to larger datasets, either from other viral pathogens or by expanding the number of HIV samples available, will allow more traditional splitting into training and test sets and increase the power of the analysis to reveal subtle patterns in the dataset. We focused our analysis on the C2-V3-C3 region of *env,* in part because of its biological relevance, but also because this region contained the best sequencing coverage. However, our method is also well suited to analysis of data sets with wider sequencing. Indeed, the iterative signature generation we utilized can be applied to identify genetic signatures across a large span of genetic sequence.

Application of modern sequencing technologies has facilitated the assembly of large datasets of viral pathogen sequences from clinical samples. As the depth and power of these datasets expands, the challenges of analyzing clinically-derived data from rapidly evolving viral pathogens across multiple hosts also increases. To fully utilize these datasets, it is imperative to design analysis techniques that can address these challenges in an efficient and robust manner. We developed a technique that uses validated data mining tools that give us the flexibility and power to increase the dimensionality of our analysis and mine the biochemical properties represented by amino acid identities. This method represents a significant advance in the ability to identify clinically important genetic signatures from sequence data sets. Its application to a variety of viral pathogens will lead to greater understanding of host-pathogen interactions. Applying this technique to HIV *env* sequences from the brain allowed us to identify genetic signatures correlated with the development of HAD. Examining the amino acid and biochemical requirements of these signatures will inform further investigations into mechanisms driving the development of HAD, with the goal of developing better diagnosis tools and treatment regimens. Further development and application of this analysis pipeline also has broader applications for the identification of genetic signatures linked to clinical outcome in other viral pathogens.

## Supporting Information

Figure S1 **Amino acid identity and biochemical factor requirements for HAD and non-HAD associated signatures.** Amino acid requirements at each position in HAD and non-HAD associated signatures are plotted. For each “position: factor” pair, all amino acids are plotted at their value for that factor. Amino acids observed at that position within the brain-derived dataset are plotted in black, while those not observed are gray. The B-clade consensus amino acid is plotted in large font. The colored bar indicates the range of acceptable values in that signature. Lower range ends are open, indicated by a dotted line, (signature 1_01, position 328 excludes Q). Upper range ends are closed, indicated by a solid line (signature 2_03, position 321 includes S).
(PDF)

Figure S2 **Proportion of sequences per patient from the brain training dataset matching HAD and non-HAD signatures.** For each signature, HAD (red) and non-HAD (blue) patients are plotted according to their total number of sequences (x-axis) and number of sequences matching the signature (y-axis). Patients with no matching sequences are omitted from the plot for clarity, but are included for statistical calculations. Dashed line indicates slope = 1 at which all sequences in a patient match signature. Jitter has been added to visualize overlapping points. Text indicates p-value by Fisher’s exact test and the number of patients from each class with matching sequences.
(PDF)

Table S1 Patient details for the brain training, brain validation, and CSF validation HIV *env* sequence datasets. All annotations are drawn from the original publication, or from the HIV Brain Sequence Database, which drew annotations from the original publication. Blanks indicate data not available.
(XLSX)
