# Supplementary figures and images for: A Machine Learning Approach for Identifying Amino Acid Signatures in the HIV Env Gene Predictive of Dementia
Source: PLoS One. 2012 Nov 14;7(11):e49538. doi: 10.1371/journal.pone.0049538 (PMC3498126; doi:10.1371/journal.pone.0049538)

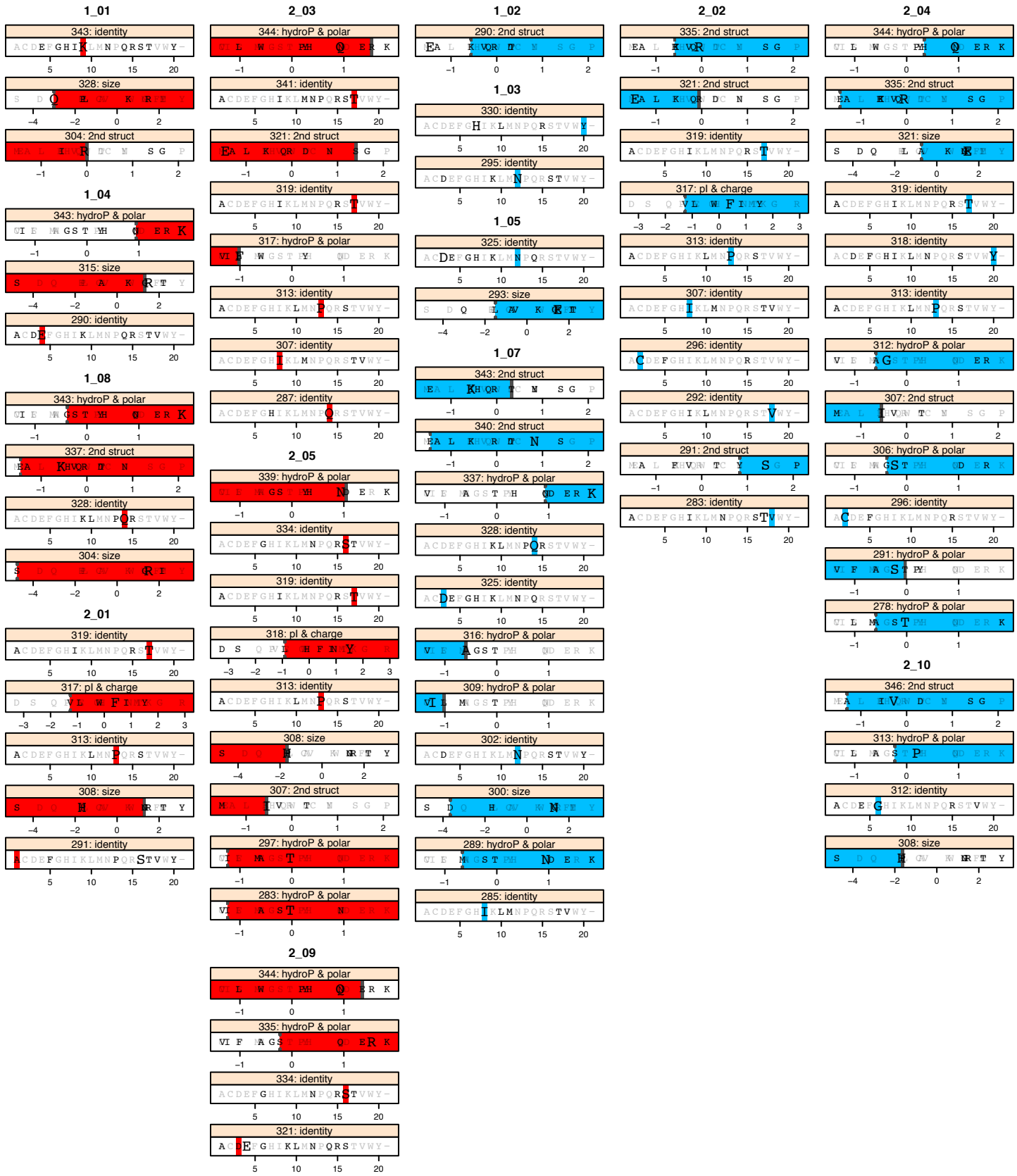

Supplement: Figure S1 — Amino acid identity and biochemical factor requirements for HAD and non-HAD associated signatures. Amino acid requirements at each position in HAD and non-HAD associated signatures are plotted. For each “position: factor” pair, all amino acids are plotted at their value for that factor. Amino acids observed at that position within the brain-derived dataset are plotted in black, while those not observed are gray. The B-clade consensus amino acid is plotted in large font. The colored bar indicates the range of acceptable values in that signature. Lower range ends are open, indicated by a dotted line, (signature 1_01, position 328 excludes Q). Upper range ends are closed, indicated by a solid line (signature 2_03, position 321 includes S). (PDF) [file pone.0049538.s001.pdf]

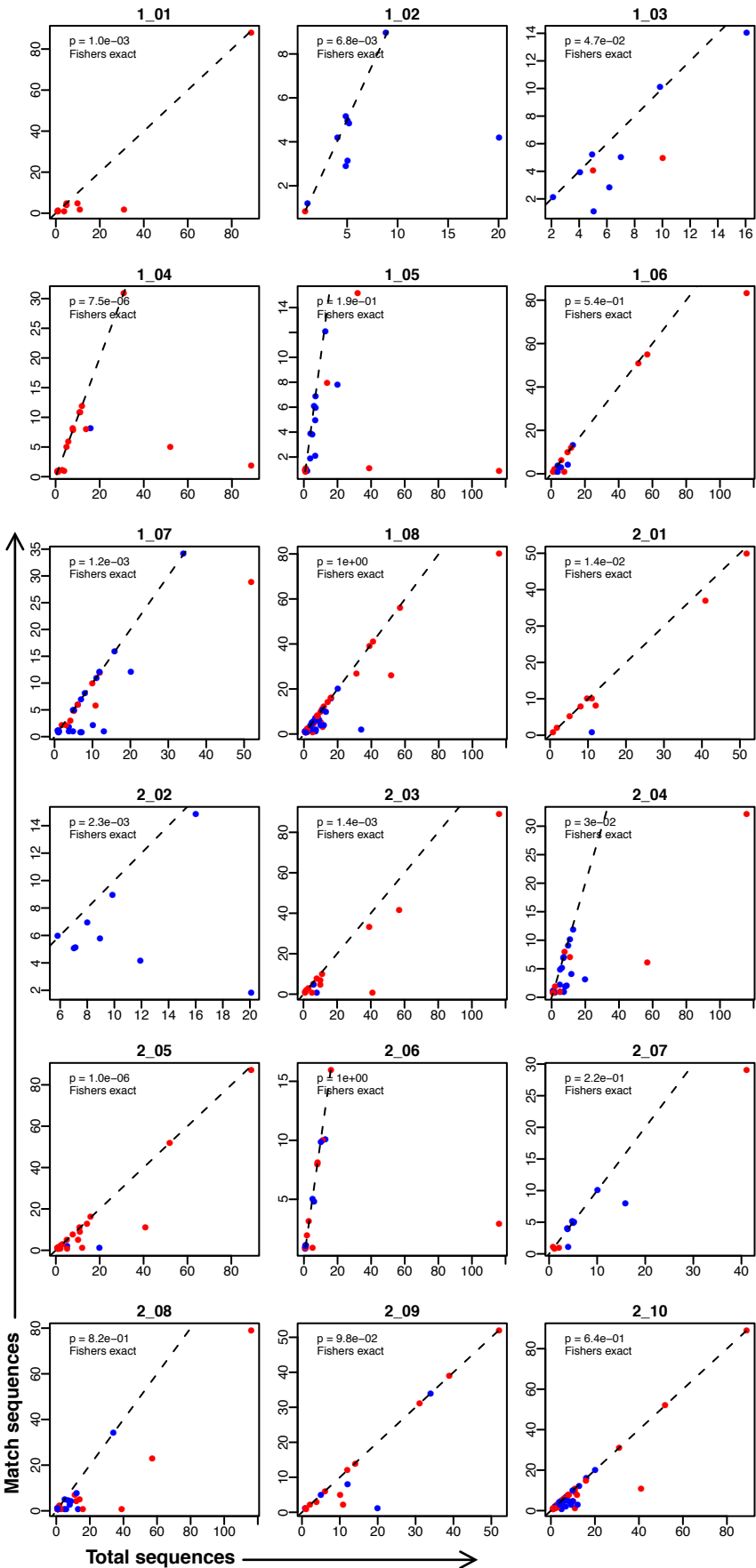

Supplement: Figure S2 — Proportion of sequences per patient from the brain training dataset matching HAD and non-HAD signatures. For each signature, HAD (red) and non-HAD (blue) patients are plotted according to their total number of sequences (x-axis) and number of sequences matching the signature (y-axis). Patients with no matching sequences are omitted from the plot for clarity, but are included for statistical calculations. Dashed line indicates slope = 1 at which all sequences in a patient match signature. Jitter has been added to visualize overlapping points. Text indicates p-value by Fisher’s exact test and the number of patients from each class with matching sequences. (PDF) [file pone.0049538.s002.pdf]
